# Clinical Applications of Hemolytic Markers in the Differential Diagnosis and Management of Hemolytic Anemia

**DOI:** 10.1155/2015/635670

**Published:** 2015-12-27

**Authors:** W. Barcellini, B. Fattizzo

**Affiliations:** U.O. Oncoematologia, Fondazione IRCCS Ca' Granda Ospedale Maggiore Policlinico di Milano, Via Francesco Sforza 35, 20100 Milano, Italy

## Abstract

Several hemolytic markers are available to guide the differential diagnosis and to monitor treatment of hemolytic conditions. They include increased reticulocytes, an indicator of marrow compensatory response, elevated lactate dehydrogenase, a marker of intravascular hemolysis, reduced haptoglobin, and unconjugated hyperbilirubinemia. The direct antiglobulin test is the cornerstone of autoimmune forms, and blood smear examination is fundamental in the diagnosis of congenital membrane defects and thrombotic microangiopathies. Marked increase of lactate dehydrogenase and hemosiderinuria are typical of intravascular hemolysis, as observed in paroxysmal nocturnal hemoglobinuria, and hyperferritinemia is associated with chronic hemolysis. Prosthetic valve replacement and stenting are also associated with intravascular and chronic hemolysis. Compensatory reticulocytosis may be inadequate/absent in case of marrow involvement, iron/vitamin deficiency, infections, or autoimmune reaction against bone marrow-precursors. Reticulocytopenia occurs in 20–40% of autoimmune hemolytic anemia cases and is a poor prognostic factor. Increased reticulocytes, lactate dehydrogenase, and bilirubin, as well as reduced haptoglobin, are observed in conditions other than hemolysis that may confound the clinical picture. Hemoglobin defines the clinical severity of hemolysis, and thrombocytopenia suggests a possible thrombotic microangiopathy or Evans' syndrome. A comprehensive clinical and laboratory evaluation is advisable for a correct diagnostic and therapeutic workup of the different hemolytic conditions.

## 1. Introduction

The term hemolysis refers to the destruction of the red blood cells (RBC) and accounts for a wide range of laboratory and clinical conditions, both physiological and pathological. It is also used to address situations in which erythrocytes half-life is diminished because of mechanical, chemical, autoimmune, or infective causes. If RBC destruction rate is high enough to determine a decrease in hemoglobin values below the normal range, hemolytic anemia occurs. This peripheral destruction/loss driven anemia, together with hemorrhage and sequestration forms, differs from anemia due to bone marrow erythrocyte production impairment (pure red cell aplasia, myelodysplasia, myelophthisis, other hematologic malignancies, and iron or vitamin deficiency) for alterations of laboratory markers, acuteness of onset, and treatment strategies.

**Figure 1:**
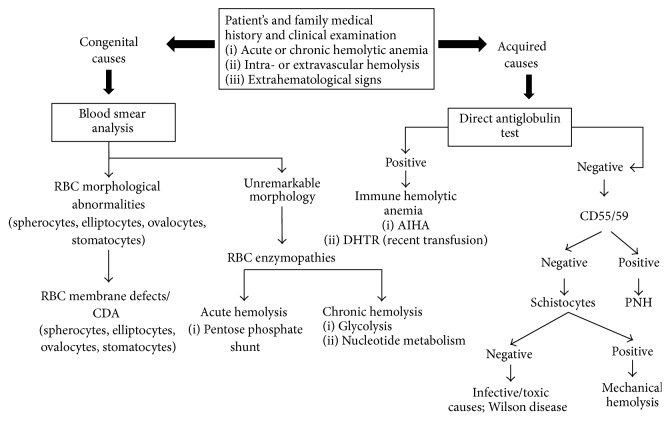
Diagnostic flowchart for hemolytic diseases. If the diagnostic flowchart turns negative for congenital hemolytic anemia, reconsider acquired causes and* vice versa*. RBC: red blood cells; AIHA: autoimmune hemolytic anemia; DHTR: delayed hemolytic transfusion reactions; CDA: congenital dyserythropoietic anemia; PNH: paroxysmal nocturnal hemoglobinuria.

A synthetic diagnostic flowchart of hemolytic anemia is shown in Figure 1 and includes firstly the distinction between congenital and acquired causes through a careful patient's and family medical history. Moreover, it is essential to distinguish the acute or chronic characteristics of anemia, the features of intra- or extravascular hemolysis, and the presence of extrahematological signs. Microscopic blood smear examination, although not routinely done nowadays, is still fundamental when performed by an expert operator and sometimes is determinant for the diagnosis, particularly in congenital forms. As regards acquired hemolytic anemia, the direct antiglobulin test (DAT) is the cornerstone for the diagnosis, enabling the distinction of autoimmune hemolytic anemia (AIHA) in warm forms (~70% of cases, DAT positive for IgG or IgG + C), cold agglutinin disease (CAD) (~20% of patients, DAT positive for C), and mixed forms (<10% of cases, DAT positive for IgG and C, with coexistence of warm autoantibodies and high titer cold agglutinins). However, there is increasing evidence of atypical cases of difficult classification, mainly DAT-negative, which are frequently severe and refractory/relapsing after several therapy lines and may have a fatal outcome [1–3]. In addition, there are rare cases caused by IgM autoantibodies with a thermal activity close to physiological temperatures (warm IgM), characterized by a severe course and a reported mortality rate of about 20% [4]. AIHA can be primary or secondary to lymphoproliferative syndromes, infections, immunodeficiency, and tumors and is described with increasing frequency following hematopoietic stem cell transplantation (HSCT). Moreover, coexisting causes of anemia can make the differential diagnosis quite challenging, and many confounders may be present at the same time, such as vitamin deficit or dysmyelopoiesis, altering the peripheral blood picture in a hemolytic patient. Furthermore, comorbidities may affect the clinical presentation, for instance, liver disease (decreased production of hemolytic markers) and renal impairment (insufficient erythropoietin production).

Several markers or hemolysis (Table 1) have been described, which are variably altered in the different forms of hemolytic anemia, thus helping in the differential diagnosis and in evaluating the efficacy of treatment. In this review, the main biochemical hemolytic markers are discussed, focusing on the differential diagnosis, the correlation with disease activity, and the response to therapy, with a particular focus on AIHA.

## 2. Hemolytic Markers

### 2.1. Hemoglobin

Hemoglobin is the most direct indicator of clinical severity in hemolytic diseases. Its levels may be close to normal values in mild forms (Hb > 10 g/dL) or importantly reduced in moderate (Hb 8–10 g/dL), severe (Hb 6–8 g/dL), and very severe (Hb 6 g/dL) forms [5]. In a recent large retrospective study of 308 cases of primary AIHA, hemoglobin values at diagnosis were the most important predictor of outcome, correlating with the risk of death and the requirement of multiple therapy lines [2]. In the differential diagnosis an acute onset is more frequently observed in RBC enzymopathies involving the pentose phosphate (PP) shunt (e.g., glucose-6-phosphate-dehydrogenase, G6PD deficit) and in autoimmune hemolytic forms involving complement activation (AIHA caused by warm IgM, warm IgG + C, mixed, and CAD with thermal range close to physiological temperatures) and in paroxysmal nocturnal hemoglobinuria (PNH). A chronic course with possible acute exacerbations is more commonly found in RBC membrane defects (e.g., hereditary spherocytosis, hereditary stomatocytosis), enzymopathies of the nucleotide and glycolytic metabolism (e.g., pyruvate-kinase deficiency), cold AIHA, and prosthetic cardiac valves or intravascular devices. A rapid decrease in hemoglobin values usually leads to relevant symptoms (e.g., asthenia, tachycardia, and dyspnea), whereas a chronic and progressive decrease is usually well tolerated. Generally, RBC destruction rate is much higher in intravascular hemolysis, calculated as 200 mL of RBC in 1 hour, whereas RBC destruction in extravascular hemolysis is 10-fold less [1].

Hemoglobin levels should be closely monitored in hemolytic patients and are pivotal for treatment response evaluation. In AIHA the response to therapy is usually defined as “complete” for Hb > 12 g/dL and hemolytic markers normalization or “partial” for Hb levels > 10 g/dL or 2 g/dL increase and reduction of hemolytic markers with no transfusion need [2, 6].

### 2.2. Reticulocytes

Reticulocytes are nonnucleated direct precursors of RBC, presenting increased mean corpuscular volume and a basophilic cytoplasm due to ribosomal-RNA traces. They represent a small fraction of peripheral RBC (normal values are about 1%; normal range may vary between laboratories).

Reticulocytes are an index of bone marrow hemopoietic activity and are usually increased in hemolysis, as well as in other pathological and physiological conditions (e.g., hemorrhage pregnancy, delivery, and acclimation). In hemolytic conditions, however, the compensatory reticulocytosis may be inadequate or absent in the presence of concomitant marrow involvement (oncohematologic conditions, dyserythropoietic or bone marrow failure syndromes), iron and vitamin deficiency, infections, or autoimmune reaction against bone marrow-precursors. The latter is of particular interest in AIHA, where reticulocytopenia is reported in 39% of children [7] and about 20% of adults [2, 8]. Reticulocytopenia may often represent a clinical emergency with an extremely high transfusion need and a poor outcome, as recently shown in 13 very severe, refractory, and fatal AIHA cases [3]. Therefore, reticulocytes should be evaluated as absolute number or with the recently proposed bone marrow responsiveness index (BMRI) [patient's absolute reticulocyte count × (patient's Hb/normal Hb)]. This index with a cutoff value of <121 is able to discriminate hemolysis with concomitant inadequate reticulocytopenia with a sensitivity of 90% and specificity of 65% in 205 patients with congenital dyserythropoietic anemia type II [9].

Reticulocytosis is an important marker to monitor recovery from hemolysis or response to specific therapy. The reticulocyte response typically requires 3 to 5 days to occur, as observed in case of supplementation with folate, B12 vitamin, or iron in patients with a deficiency (the so-called reticulocyte crisis). In AIHA reticulocytes usually remain elevated for several days until hemoglobin levels are restored; in patients with inadequate reticulocytosis, erythropoietin has been shown to improve anemia and to reduce/avoid hemolysis related to overtransfusion, as observed with thrombopoietin agonists in primary immune thrombocytopenia [2, 10]. In chronic/congenital hemolytic conditions, reticulocytes are usually mildly elevated but can dramatically rise in acute hemolytic crisis. In hereditary spherocytosis absolute reticulocyte counts significantly decrease after splenectomy, consistently with the reduced hemolytic condition [11]. At variance, this does not occur in pyruvate-kinase deficit, where a persistent increased reticulocyte count is observed after splenectomy [12]. Likewise, in patients with PNH, the reticulocyte counts often remain elevated during treatment with eculizumab, because of the persistence of some extravascular hemolysis due to deposition of C3 fragments on PNH red cells [13]. Finally, reticulocyte count does not significantly change in patients with prosthetic valve replacement, as this condition usually implies subclinical hemolysis with normal or slightly decreased hemoglobin levels [14].

### 2.3. Schistocytes

A schistocyte is a fragmented part of a RBC, which is visible at peripheral blood smear as an irregularly shaped body with two pointed ends without central pallor. Schistocytes derive from a mechanical fragmentation of RBC due to an obstacle within the vessels, such as fibrin clots, mechanical artificial heart valve, or any other intravascular devices. In healthy individuals, normal schistocyte count is below 0.5%. A count superior to 1% is typical of thrombotic thrombocytopenic purpura (TTP) with a common range of 3–10%, whereas a value between 0.5% and 1% is suggestive of disseminated intravascular coagulation (DIC). Once intravascular devices and DIC are excluded, the differential diagnosis encompasses thrombotic microangiopathies, caused by hemostasis activation in the small vessels with consumption of platelets, coagulation factors, and RBC.

In TTP, aberrant hemostasis occurs because of congenital or acquired deficiency of ADAMTS 13, a metalloproteinase responsible for von Willebrand multimers degradation. Other causes of thrombotic microangiopathies are typical hemolytic uremic syndrome (HUS), due to Shiga-like toxin activity, and atypical HUS, caused by aberrant complement activation. Because in these diseases an intravascular hemolysis occurs LDH values are usually increased. Moreover, in a pregnant woman with hemolytic anemia, arterial blood pressure, platelets levels, and liver enzymes should be evaluated as hemolysis with elevation of liver enzymes and low platelets (HELLP) syndrome may occur. Other important features for the differential diagnosis are proteinuria and renal impairment (more pronounced in HELLP, typical and atypical HUS), undetectable ADAMTS 13 activity and neurological symptoms (common in TTP), and history of* E. coli* diarrhea (hallmark of typical HUS) [15]. Blood smear examination should be early performed to detect schistocytes if microangiopathic hemolytic anemia is suspected. In fact, a prompt treatment of these diseases within the first hours from the diagnosis dramatically decreases their mortality. This is observed in TTP, where mortality is reduced to approximately 10% if large volume plasma exchange (60 mL/Kg) is started as soon as the diagnosis is suspected and continued every day until platelets recovery. The same has been demonstrated for atypical HUS, where eculizumab may be used, and for HELLP, which readily improves after delivery induction [15, 16].

### 2.4. Lactate Dehydrogenase

Lactate dehydrogenase (LDH) is an enzyme that catalyzes the conversion of lactate into pyruvic acid, located in cytoplasm and distributed in various organs (e.g., heart, muscle, liver, and brain). LDH is physiologically measurable in serum due to physiological cellular turnover and 5 isoenzymes are present. In particular, LDH-1 and LDH-2 isoenzymes are expressed in RBC. In the hemolytic conditions, LDH (mainly isoenzymes 1 and 2) is often increased and may be useful to distinguish extravascular versus intravascular hemolysis, being slightly increased in the former (e.g., warm AIHA and congenital forms) and 4-5-fold the upper normal limit in the latter (e.g., PNH, prosthetic valve hemolysis). In patients with AIHA, higher LDH levels were observed in warm IgG + C and cold forms with a thermal range close to 37°C, where intravascular hemolysis is due to complement activation and correlates with clinical severity and thrombotic events [2].

Moreover, in patients undergoing prosthetic valve replacement, LDH was significantly more increased in patients with mechanic than biologic prosthetic valve and in those with double than single valve replacement [14]. Finally, it is worth reminding that LDH may significantly rise in case of acute hemolytic crisis due to infections in patients with congenital hemolytic anemia.

LDH is useful in evaluating response to treatment, as its level decreases along with the reduction of the hemolytic rate. This has been described in AIHA, atypical HUS and PNH following therapy [1, 17], and microangiopathic hemolytic anemia after plasma exchange [18]. Moreover, in patients with PNH under eculizumab, breakthrough intravascular hemolysis and a return of PNH symptoms may typically occur 1 or 2 days before the next scheduled dose. This is associated with a spike in the LDH level, suggesting shortening the interval between administrations or an increase in eculizumab dose [17]. Because of its large distribution, LDH can increase in several conditions other than hemolysis, which involve cellular necrosis and increased tissue turnover (e.g., myocardial infarction, heart failure, hepatitis of all etiologies, extreme muscular effort, and solid and hematologic tumors). Very recently, a ratio between LDH and aspartate aminotransferase above 22.12 has been shown to differentiate TTP from other thrombotic microangiopathies (e.g., HUS and HELLP syndrome) even before ADAMTS 13 activity test results [15]. Moreover, LDH levels may be markedly increased in patients with vitamin B12 or folic acid deficiency, because of ineffective erythropoiesis and premature RBC death.

### 2.5. Haptoglobin

Haptoglobin (Hp) is a glycoprotein synthetized by the liver sorting within alpha-2 globulins at serum electrophoresis. It has antioxidant and immunomodulatory properties [19] and acts as a scavenger by stably binding free serum circulating hemoglobin released by hemolysis or normal RBC turnover. The resulting complexes are promptly cleared by reticuloendothelial system via CD163 receptors, preventing the generation of reactive oxygen species and renal damage. After endocytosis, the haptoglobin-hemoglobin complex is degraded by lysosomes resulting in haptoglobin depletion [20]. Haptoglobin is not a homogeneous protein as there are two common alleles, called Hp1 and Hp2. This leads to three possible variants (1-1, 2-2, and 1-2) with different molecular weight polymers, distinguishable at high resolution electrophoresis, which show different behavior in their shielding against oxidative stress [21].

Haptoglobin is significantly decreased during hemolysis, [22] both in intravascular forms, due to increased free plasma Hb and altered free/complexed haptoglobin balance, and in extravascular cases, where little intravascular lysis of structurally altered RBC escaped from reticuloendothelial clearance may be present [23]. In AIHA haptoglobin represents the most sensitive marker of hemolysis and it is the last one to normalize after recovery, possibly remaining decreased even in the presence of normal Hb levels (personal observation).

Concerning the differential diagnosis, reduced levels of haptoglobin are observed in conditions other than hemolysis, such as liver impairment, malnutrition, and congenital hypohaptoglobinemia. On the other hand, haptoglobin increases in inflammatory diseases, in cigarette smokers, and in nephrotic syndrome, possibly masking an underlying hemolytic condition. In a study of 100 patients with a variety of hematologic and nonhematologic diseases, a haptoglobin limit of 25 mg/dL or less allowed the identification of hemolytic from nonhemolytic disorders with a sensitivity and specificity of 83% and 96%, respectively [24].

### 2.6. Bilirubin

Bilirubin IXa derives from the catabolism of the protoporphyrin IX ring of heme, the prosthetic group of proteins involved in oxygen transport and metabolism (e.g., Hb, myoglobin, and cytochrome P450) by microsomal heme-oxygenase. 85% of the circulating bilirubin derives from hemoglobin catabolism in the reticuloendothelial organs. Ineffective erythropoiesis in the bone marrow, which is present at low rate in physiological conditions, constitutes an additional bilirubin source. Bilirubin is a good marker for extravascular and, to a lesser extent, also for intravascular hemolysis, where a minor fraction of the released heme binds to hemopexin and undergoes reticuloendothelial catabolism in the liver. Bilirubin, produced in the periphery, is transported to the liver tightly bound to albumin (namely, unconjugated bilirubin). In the liver bilirubin is converted to bilirubin mono- and diglucuronides by the microsomal enzyme bilirubin UDP-glucuronosyltransferase and then is excreted with the bile (conjugated bilirubin). Increased bilirubin levels may therefore be due to increased hemoglobin catabolism (mainly resulting in unconjugated hyperbilirubinemia) or to decreased hepatic clearance (most commonly conjugated hyperbilirubinemia). Bilirubin upper normal limit should be corrected for variations in the circulating RBC mass, by dividing patient's hematocrit by 45, so that in patients with reduced hematocrit a little bilirubin increase may already be considered pathological. Hyperbilirubinemia during hemolysis is usually no more than 4 mg/dL, and higher values almost always imply a concomitant reduction in the hepatic function, which can be easily investigated by liver functional markers. Unconjugated values greater than 4 mg/dL are however observed in acute massive hemolysis (e.g., G6PD deficiency or transfusion reactions) [25] and in the case of coexisting Gilbert's syndrome, reported in 5% of the general population and characterized by a reduction in the activity of the hepatic bilirubin UDP-glucuronosyltransferase [26].

Bilirubin is an early marker of therapy response as it returns to normal or within 10% of normal values in 4 hours after hemolysis cessation. In AIHA a reduction of unconjugated bilirubin concentration is observed already on the 7th day of steroid therapy, providing early evidence of therapeutic response [25]. Moreover, levels of unconjugated bilirubin significantly decreased after splenectomy in hereditary spherocytosis [11], and total circulating bilirubin tends to decrease in patients with PNH treated with eculizumab [17].

### 2.7. Ferritin

Ferritin is an intracellular protein that stores iron and releases it in case of request, acting as a buffer against iron deficiency and iron overload. It may be used as an indirect marker of the total body amount of iron. Ferritin is increased in several chronic hemolytic conditions, such as congenital membrane defects and enzymopathies, chronic cold agglutinin disease, and congenital dyserythropoietic anemia [9, 11, 12]. Although the precise mechanism leading to its increase has not been investigated, it has been hypothesized that iron produced by ineffective erythropoiesis and extravascular hemolysis is not easily eliminated and that anemia itself is a powerful stimulus for iron absorption in the gut. Patients with PNH may display either increased ferritin values when under eculizumab treatment probably because of continuous extravascular hemolysis (personal observation) or reduced ferritin levels due to hemosiderinuria and iron loss [17]. Ferritin is an acute phase protein and increases in various metabolic and inflammatory diseases (e.g., chronic and acute infections, hepatitis, and tumors). Thus the coexistence of these conditions together with chronic hemolysis may give rise to particularly high ferritin values. Moreover iron overload is observed after transfusion and in patients with hereditary hemochromatosis. The presence of an undiagnosed heterozygous condition for hemochromatosis may further increase hyperferritinemia due to chronic hemolysis. In hereditary spherocytosis a serum ferritin concentration > 500 ng/mL was detected in 8 out of 189 nonsplenectomized and never transfused patients, and 3 of them were found to have the hemochromatosis His63>Asp mutation in heterozygosity [11]. Finally, transfusion support, which is often given to hemolytic patients, may also contribute to iron overload.

### 2.8. Other Complete Blood Count Abnormalities

Among the several alterations of blood counts observed in the different hemolytic diseases considered, the possible reduced leukocytes and platelets in B12 and folic acid deficiency are worth reminding. Moreover, the presence of thrombocytopenia suggests a possible thrombotic microangiopathy or Evans' syndrome. The latter should be promptly recognized and treated, as it has been recently reported as a negative prognostic factor in patients with AIHA [2]. Leukocytes and platelets may also be slightly reduced in patients with PNH, as these cells are deficient for CD55/CD59 molecules and they may be damaged by complement activation. More pronounced leucopenia and thrombocytopenia occur in case of PNH-associated bone marrow failure syndromes. In patients with congenital membrane or enzyme defects mild thrombocytopenia may be related to hypersplenism, whereas thrombocytosis may be observed after splenectomy. Finally, platelets and leukocytes may also be destroyed by intravascular devices.

### 2.9. Hemosiderinuria

Hemosiderinuria is the presence of hemosiderin bound to iron in the urine and accounts for the “brownish” color of urine, typically associated with marked intravascular hemolysis. Hemoglobin released into the bloodstream in excess of haptoglobin binding capacity is filtered by the kidney and reabsorbed in the proximal convoluted tubule. Here, the iron portion is removed and stored as ferritin or hemosiderin and then excreted into the urine. Hemosiderinuria is typically observed in PNH, incompatible RBC transfusion, G6PD deficiency, severe burns, and infections. It is usually seen 3-4 days after the onset of hemolysis and it can persist for several weeks after hemolysis cessation, whereas hemoglobinuria quickly disappears.

### 2.10. Treatment of AIHA

The distinction between warm AIHA and CAD is fundamental, as the two forms have quite different responses to the available therapies. For warm AIHA steroids represent the first-line therapy [1]: oral prednisone is usually given at 1–1.5 mg/kg/day for 1–3 weeks until hemoglobin >10 g/dL and then slowly tapered off over a period not shorter than 4–6 months. Intravenous methylprednisolone 100–200 mg/day for 10–14 days or 250–1000 mg/day for 1–3 days may be indicated in very severe or complex cases such as Evans' syndrome. Steroids are able to provide a response in 70–85% of patients, but with an estimated cure rate in 20–30% only. Second-line treatment for warm forms includes splenectomy [27] (early response rate in ~70% and a presumed curative rate in ~20% of cases but associated with infective and thrombotic risk) and rituximab, which is increasingly preferred (response rate of about 70–80% and disease-free survival of ~70% at one and ~55% at two years) [28]. Conventional immunosuppressive drugs (such as azathioprine, cyclophosphamide, and cyclosporine) are mostly used as steroid-sparing agents when splenectomy is not feasible and/or rituximab unavailable. Response rates are reported in 40–60% but partially attributable to concomitant steroid administration, and serious side effects are not infrequent [1, 27]. Further treatments include mycophenolate and for the few ultra-refractory patients high dose cyclophosphamide, alemtuzumab, plasma exchange, and erythropoietin, particularly in the presence of reticulocytopenia. Cold agglutinin disease deserves treatment in the presence of symptomatic anemia, transfusion dependence, and/or disabling circulatory symptoms. Steroids are now discouraged, as they are effective at unacceptably high doses and in a small fraction of cases (14–35%), and splenectomy is usually unsuccessful [1, 29]. Rituximab is now recommended as the first-line treatment, being effective in ~60% of cases (5–10% complete responses), with a response duration of 1-2 years [30]. For refractory/relapsing cases other options are rituximab plus fludarabine, bortezomib, and eculizumab, although further studies are required to confirm their efficacy. Future promising drugs are the new complement inhibitors TNT003, C1-esterase inhibitors, compstatin Cp40, and TT30 [31].

## 3. Conclusion

Hemolytic anemia contains a group of heterogeneous diseases both congenital and acquired in which the diagnosis may be challenging. Blood smear examination is still fundamental, and DAT is the cornerstone for the diagnostic workup of acquired forms but may be affected by various drawbacks. Hemolytic parameters may be differently altered in the various conditions thus helping the differential diagnosis. However, many confounders may further complicate the clinical picture, underlining the need for a comprehensive clinical and laboratory evaluation. Finally, hemolytic markers are undoubtedly important in monitoring the efficacy of treatment.

## Figures and Tables

**Table 1:** Markers of hemolysis in different hemolytic diseases.

	AIHA	Membrane/enzyme defects	CDA	PNH	TMA	Intravascular devices
Hb	− to − − −	−/− −	− −/− − −	− −/− − −	− −/− − −	−
Reticulocytes	− to +++	+ to +++	−/=	− to ++	+	+
Schistocytes	=	=	=	=	++	+
LDH	+/++	+	+	+++	++	++
Haptoglobin	− − −	− − −	− −	− − −	−	− −
Bilirubin	+	++	+	+	+	+
Ferritin	=/+	++	+++	− to +	=/+	=/+
PLT	=/− −	=/−	=	=/−	− −	=/−
WBC	=	=	=	=/−	=	=/−
Hemosiderinuria	=/+	=	=	+ to +++	=/+	=/+

Values are expressed in a semiquantitative style to indicate the different intensity of alteration in the various hemolytic syndromes, as follows: +/++/+++ indicate an increase from mild to severe, −/− −/− − − indicate a reduction, and = indicates values within the normal range.

AIHA: autoimmune hemolytic anemia; CDA: congenital dyserythropoietic anemia; PNH: paroxysmal nocturnal hemoglobinuria; TMA: thrombotic microangiopathies; Hb: hemoglobin; LDH: lactate dehydrogenase; PLT: platelets; WBC: white blood cells.
